# Social construction of disability and its potential impacts to welfare practice in Vietnamese contexts

**DOI:** 10.1186/2193-1801-3-325

**Published:** 2014-06-28

**Authors:** Kham V Tran

**Affiliations:** University of Social Sciences and Humanities, Vietnam National University, Hanoi, Vietnam

**Keywords:** Vietnam, Disability, Social construction, Social welfare, Children with disabilities

## Abstract

From the survey responses and the policy analysis, the initial findings on this paper present some aspects of knowledge, attitude and practice (KAP) on disability which are presented as following: *Firstly*, there is a significant changes in legal documents and social policies related to disability in Vietnam, especially from 2006, in terms of its name and contents for improving the life of PWD with inclusive approach, however the meaning of disability is not clear in policies. *Secondly*, the understanding on disability is mainly based on medical/individual model which focuses on the disability’s causes in words of health or individual problem rather than viewing the social causes in aspects of the social barriers and restriction, in addition almost policies focus on the problems of PWD rather than the social aspects. *Thirdly*, social attitude toward disability and PWD seems to be very empathetic, however it is less regard to CWD’s ability as well as there are more attitudes on charity giving and supporting than helping them to be independent in their life. Finally, in spite of positive knowledge and attitudes on disability, there is still limitation on practical activities towards CWD/PWD from society in daily life.

## Introduction

Recent statistics by Vietnam Ministry of Labours, Invalids and Social Affairs (MOLISA) identify that there is more than 6.7 million people with disabilities (PWD) or more than 6.34% of the population of Vietnam (MOLISA 2004; UNICEF Vietnam 2010). Vietnamese Government tries to setup a variety of legal documents and social policies in order to promote the life of PWD. However, they are still living in poor conditions and facing negative social attitudes as well as experience their own difficulties in accessing the social supports from welfare systems (MOLISA 2004; Tran 2014; UNICEF Vietnam 2010). The welfare policies toward PWD has been changed but there are still limited in providing sustainable services and supports for PWD and their families as well as creating an inclusive setting for their social participation.

Reasons for such situations are expressed as the lack of social awareness on disability; the uncomprehensive understandings of social position of PWD in society; the existed social policies are not effective in practice and in creating the specific services as well (Le et al. 2008; The United States Agency for International Development 2005). Based on social constructionism as theoretical approach, it is found that the meaning of disability and its social understanding are very significant for changing the social attitudes toward PWD and for changing the way of delivering social supports for them (Tran 2013). This situation leads to require more considerations in social research about the social construction of disability in Vietnamese contexts, in both policy approaches and social understandings. Having the details of such understandings also creates the significant impact to welfare practice to the life of PWD and also improves the social inclusion aspect for PWD. This also aims at mapping the harmonised and sustainable society for all (UNICEF Vietnam 2010).

This paper focuses on the general understanding and practice on disability from policy analysis and surveys as the exploratory research to understand the contents of disability in Vietnam contexts. Focusing on the analysis of policy and the daily understandings in disability is the way to identify the gaps between the policy and practice in area of disability. This work paper aims at making the recommendation for promoting social inclusion of CWD in Vietnam in ideas of social welfare reform and social work practice.

From the survey with 230 participants and the policy analysis, the initial findings on this paper present some aspects of knowledge, attitude and practice (KAP) on disability which are presented as following: *Firstly*, there is a significant changes in legal documents and social policies related to disability in Vietnam, especially from 2006, in terms of its name, its contents for improving the life of PWD with inclusive approach, however the meaning of disability is not clear in policies. *Secondly*, the understanding on disability is mainly based on medical/individual model which focuses on the disability’s causes in words of health or individual problem rather than viewing the social causes in aspects of the social barriers and restriction, and almost policies focus on the problems of PWD rather than the social aspects. *Thirdly*, social attitude toward disability and PWD seems to be very empathetic, however it is less regard to CWD’s ability as well as there are more attitudes on charity giving and supporting than helping them to be independent in their life. Finally, in spite of positive knowledge and attitudes on disability, there is still limitation on practical activities towards CWD/PWD from society in daily life.

## Methods

Methods for collecting and generating data and implications for satisfying the research aims in this paper, based on the initial outcomes of the research on children with disabilities in Hanoi-Vietnam, consist of social analysis and survey. They are two main methods on the research process, based on the model of Crotty, which includes four significant elements as: Epistemology, theoretical perspective, methodology and research method (Crotty 1998).

### Document analysis

Legal documents and social policies, in areas of disability since 1986, are analysed with two main aspects: The name of disability and its contents relating to the life of PWD in terms of education, health care, employment, transportation and accessibility.

### Survey

Survey’s research populations are included as: CWD, CWND in mainstream schools, parents of CWD, teachers and community persons who experience their life with CWD. The questionnaires are delivered from school to families with CWD and those people living around CWD’s houses. In order to make the simplicity of survey data, research participants are grouped into PWD and PWND.

There are three parts on the survey. The first part consists of 7 questions on general information. The second part includes 3 main questions in terms of knowledge, awareness and practice toward disability. Inaddition, the third one has 5 questions on daily activities experienced by CWD. Research participants, including CWD, children with non-disabilities (CWND), teachers, parents of children with/without disabilities, are chosen in mainstream schools in one district of Hanoi, Vietnam. They are free to attend this research. This research focuses only CWD in types of mobility and vision impairment. For those CWD in term of visionary, the researcher reads aloud the content of survey and write-down the answers. The total number of research participants is 230.

Written informed consent was obtained from research participants for conducting the research and having publication of the research report. The research ethics is followed the approval by Vietnam National University-Hanoi, number QG.14.38.

## Findings

### Social construction of disability in Vietnamese legal documents and social policies

Following the United Nations’ Convention on the Rights of Person, Vietnam approved the Law on Disabilities, 2010 (Vietnam National Assembly 2010). And it is found that there has been a fruitful legal system on disability and PWD at present, which regard to the rights to have equal opportunities and access to health, education and jobs (MOLISA 2004). These documents in Vietnam are made on the basis of international accords and conventions such as the UN Convention of the Rights of the Child (UNICEF 1989); Salamanca Statement and Framework for Action on Special Needs Educations (UNESCO 1984); and the UN Convention on the Rights of Persons with Disabilities (United Nations 2006). The social construction of disability is understood in following aspects:

#### The name of disability

Among the available legal documents in Law Database released by the Vietnam National Assembly’s Office (http://vietlaw.gov.vn), there are differences in the name of documents in terms of disability and impairment in Vietnamese. The word “tàn tật” implied its meanings as the impairment while the word “khuyết tật” has its meaning as “disability”. Searching with “khuyết tật” is given with only two findings, one approved in 2009 and one in 2010, while the findings for “tàn tật” present 16 findings. Among the latter findings, there are 3 in 2007, 4 in 2006, and the 9 others in the period 1992 to 2005.

It is found that the signal for changes of social awareness on disability was started from 2009, at that time the Vietnamese Government and its ministries started to draft the first Law on PWD. It’s also interested that Vietnam did sign the UN’s Convention on the rights for PWD in 2006 however the changes on the understandings on disability in term of its name in policies were clearly appeared since 2009. While searching and comparing these words in http://thuvienphapluat.vn, one of the sites providing legal documents in all aspects, it is found that the total numbers of law and legal documents with the name of Impaired (tàn tật) and Disability (khuyết tật) in their titles are 95, in which 62 are belong to the group I (tàn tật) and the rests are for group II (khuyết tật) (Table 1).

**Table 1:** **Number of legal documents in term of Tàn Tật and Khuyết tật in Vietnam (from 1986 to 2013)**

	From 1986 to 2002	2003	2004	2005	2006	2007	2008	2009	2010 to 2013
**I**	23	3	2	3	8	10	8	5	1
**II**	0	2	0	2	2	4	5	6	30
**Total**	23	5	2	5	10	14	13	11	31

Note: (I) number of legal documents with the name of “Tàn Tật” in their titles.

(II) number of legal documents with the name of “Khuyết tật” in their titles.

The high density of these documents on both impairment and disability is around the period from to 2009. It is found that there is a great impact from the international conventions on the Rights for PWD, in which Vietnam signed in 2006 but it is still not ratified. Furthermore, this period was also the time the Vietnamese government and its ministries tried to draft and approve the Law on PWD (Vietnam National Assembly 2010). Among these documents, the definition of disability was not identified only ideas about the PWD and types of PWD are made from the International legal documents.

The name of disability was appeared in the legal documents since 2003 while the concept of impairment was mostly disappeared from 2009, one year before the approval of the law on PWD and it was also meant that the later term was still existed for 3 years in Vietnamese context after signing the UN’s convention on the rights of PWD. Changing the way to label the implications of disability from “Tàn Tật” to “Khuyết tật” also confirmed the State efforts on applying the international and regional documents in practice as well as changing in the social awareness on disability positively.

#### Education

This is the first priority on policies on area of disability in Vietnam. There are great numbers of policies on support for PWD in terms of: assessing to education, the rights of institutions and people involved in providing education for PWD and regulations on the suitable forms of education for PWD. The most important laws and policies include the Law on Education (Vietnam National Assembly 2005a), the decision on inclusive education for PWD, and Law on PWD (Vietnam National Assembly 2010) as well.

These policies stated the responsibilities of the State, organisations, families and individuals on education for PWD (Vietnam National Assembly 2005a:23). They also confirmed education as the main method for changing the life of PWD.

As the results, the education for PWD is formed with three modules: inclusive education, special education and integrated/semi-inclusive education. The third form is known as community based schools and classes at communes, mixed classes, schools with village-based classes, ethnic boarding schools, flexible classes, love classes which are formed to serve the needs for care, education and rehabilitation for PWD and children with special needs. It is stated that education for PWD is still limited in aspects of quantity and quality. The rate for mobilising CWD going to school is quite low, just around 28% and at low level, mainly at kindergarten and primary level. The number of special institutions is slightly increased, which is still insufficient and low quality. Recently, the State and related ministries and organisations have paid more attentions on training skills for teachers and providing more facilities in inclusive education.

#### Vocational training and employment

Vocational training and employment are important contents for creating and promoting jobs for PWD and the significant ways for them to gradually and sustainably integrate into the community (National Coordinating Council on Disability 2010). The significant policies in this section are the Labour Code (Vietnam National Assembly 2002), the Ordinance on PWD (Vietnam National Assembly 1998) and the Law on PWD (Vietnam National Assembly 2010).

The main ideas of policies on vocational training and employments are stated in identifying the minimum proportion of the workforce with disabilities in each company or organization; defining legal provisions on vocational training, creating job for PWD, support for enterprises and policies on recruiting PWD in work force. There are more specific requirements on training and creating job for PWD as well as creating good conditions for not only PWD, but also for PWND and the enterprises.

Changes on vocational training and employment for PWD has been acknowledged and progressed but the rate of PWD being trained with career skills and being recruited into workforce is still low. There is a big gap between the directions on policy and the practice, especially on aspect of recruiting and preparing the workplace for PWD and PWND.

#### Health care

In Vietnam, health care for PWD is identified as the responsibility of the Ministry of Health in the collaboration with related ministries and branches to delivering specific programs. Currently, there are some significant policies in this area as Community based functional rehabilitation strategy, Law on health insurance (Vietnam National Assembly 2008), the law on PWD (Vietnam National Assembly 2010) and wide ranges of specific policies by Ministry of Health. The main ideas from these documents are aimed at refining the criteria for classifying types of disability and levels of disability severity; implementing community based rehabilitation; improving staff’s expertise about functional rehabilitation, particularly for those on working at local health care providers; developing services of early identification and intervention for CWD.

The outputs of these polices are positive as 100% PWD in low-income are provided with health insurance cards, nearly 300 thousand PWD are supported with orthopaedic and functional rehabilitation and assisted devices as wheelchairs, pushchairs, artificial limbs (National Coordinating Council on Disability 2010). In terms of financial supports for health check and functional rehabilitation, 53.4% of PWD have been entitled. With social development, the health care and social protection for PWD is being properly concerned with aims at health care supports and developing services for early identification and intervention for PWD. However, PWD are still limited with accessibility to health services, especially in rural and remote areas or the problems of service quality, only 46% of dispensaries is satisfied with health care supports (National Coordinating Council on Disability 2010).

#### Social protection and social assistance

The Vietnam National Assembly approved the Law on protecting, caring and educating children in 2004 which is a specific law outlining the responsibilities of individual, family, organisation and society in protecting, caring and educating children. It also defines the roles and responsibilities of NGOs in Vietnam on taking care children (Vietnam National Assembly 2004). Policies on social assistance are included in two categories: regular and relief assistance. These policies are significant for supporting vulnerable groups to stabilise their lives and improve their ability on risk prevention and resistance. They are identified in the areas of supporting PWD who lost their working capacity on using the State budget, in this approach the socialising assistance was broadened and resulted in partial contribution of finance to the limited State budget.

Recent reports stated that monthly finance assistance has been provided to nearly 400,000 PWD and nearly 9000 households with 2 PWD or more (MOLISA 2009; National Coordinating Council on Disability 2010). It is increased 4 times in comparison to that numbers of entitled PWD in 1998. Other social assistances for PWD in the categories of war related as veterans and Agent Orange’s victims are also provided for up to a million people monthly. And the social assistance establishments have been increased in the number. By the end of 2008, there were 572 units across the country with the host of 14,613 persons (National Coordinating Council on Disability 2010).

In this aspect, not all PWD are benefited from these social policies, just only those in severe or in poverty conditions. This situation is existed due to the limited State budget, as well as there is lack of specific identification of disability and the methods on disability registration as well.

#### Cultural and sport activities

Legal documents on culture, sports and entertainment have been stipulated to create preferential conditions for PWD to participate in, to receive training in, to compete and develop their talents in sports, culture and arts as well as to enjoy cultural and sport values. Major legal documents to be mentioned include Law on Physical Training and Sports (Vietnam National Assembly 2006a) and The Law on PWD (Vietnam National Assembly 2010) as well.

Recent research stated that PWD are still hidden from sport and cultural activities and there is also lack of activities for PWD (Le et al. 2008; National Coordinating Council on Disability 2010; UNICEF Vietnam 2010). The causes for this situation is in term of lacking of spaces and suitable activities for PWD, almost sport facilities in communities are inadequate, not comprehensive and not suitable for PWD. Almost public cultural facilities such as cinemas, theatres and libraries are hard for PWD to access.

#### Transportation and public accessibility

To support PWD to get access to public transportation and infrastructure, the government has stipulated various legal documents that regulate priorities given to PWD joining traffic. The legislation also specifies standards by which newly constructed, upgraded and renovated transportation works ensure access by PWD. Roadway Traffic Law (Vietnam National Assembly 2008), Railway Law (Vietnam National Assembly 2005b), Vietnam Civil Aviation Law (Vietnam National Assembly 2006b) all regulate privileges given to PWD joining the transportation. They also claim that newly constructed, upgraded and renovated transportation works have to conform to required technical specifications and conditions for safe transportation of people and vehicles, including pedestrians and PWD.

The codes and standards in construction for PWD (2002) are applied to new construction and renovation of public buildings, houses, apartment buildings, roads and sidewalks. A large number of documents include the construction standards to ensure that PWD can access and use public buildings and spaces in their daily activities.

Outcomes from these legal and polices are presented in aspects of awareness-raising which made positive impacts on transportation awareness, attitude and behaviour of the community and ensured the technical specifications in constructing, upgrading and renovating transportation works and means. Changes in the reality are quiet slow, only few accessible bus routes are put into operations in some main cities in Vietnam with the free fare for PWD, and only new buildings in big cities are constructed with accessible ways and facilities. There are also lack of accessible public spaces and facilities in almost urban areas.

#### Summary

Disability is socially constructed in the Vietnamese legal and policy documents. The main contents for disability are overall for the aspects of the daily life however there are more concerns and investments on the areas of education health care and social supports. There are also specific efforts in making the changes on social settings for them but with limitations on state budgets and specific services and solutions. Disability is not clearly stated in almost legal and policy documents, only the term of people with disabilities was expressed and there is lack of clear classifications of the type and levels of disabilities as well. In the current contexts, with limitations and financial supports and professional activities, the understanding on disability is in the side of the individual model rather than in that of social one.

### Social construction of disability: survey findings

There are two main questions on this survey section; the first main question is about ideas of research participants on general understanding of disability and the second focuses on the inclusion aspect of daily life of CWD at different social contexts. The disability is identified in its own definition or from ideas on status of CWD in their life. Such formations are also applied to the other two main questions on attitudes and practice toward disability and CWD. Findings from these questions are grouped into the same types of knowledge, attitudes and practice. Structure of this survey is designed in form of knowledge, attitude and practice (KAP), investigating the respondents’ knowledge, attitudes and practices on disability itself as well as the life of PWD. All questions, in aspect of disability, are designed as the open-ended ones with Likert Scale’s style. They are grouped into 3 main questions with 35 sub-questions relating to the KAP on disability.

#### Knowledge on disability

Knowledge on disability is explored in terms of causes, ability of CWD and looking at the limitations of CWD in daily life. Being disabled due to illness, premature birth or by accident are highly acknowledged more than those causes in terms of cultural aspect (in words of cursed family, deserving to family conditions or did bad things in the past). These statements are scored with positive awareness on disability which is the same with MOLISA survey on this aspect of disability in 2000 (MOLISA 2004) as well as CRS’s research in inclusive education in Vietnam (Clarke 2006). However, in statement of “the parents have done something bad in their life”, 5.7% of respondents agreed with which is the highest response on those statements relating to cultural belief on being disabled situation. By looking at the crosstab of “PWD and PWND”, it is found that such disagreement of PWND is higher than that of PWD (77.4% in comparison with 68.4%) in these statements.

Many disability research projects suggested that researchers should concentrate on the abilities rather than the disabilities (Barton and Oliver 1997; Le et al. 2008; Vietnam Government 2010). So, in this survey, another aspect of knowledge on disability is explored in term of abilities of CWD/PWD which are the focuses of statements: *CWD can learn in same classroom with CWND*, *CWD are able to be trained in most vocational skills and CWD can learn at same rate as CWND*. Almost respondents agreed to these statements with high percentage: 84.3%, 94.3% and 81.4% respectively. These outcomes demonstrate that there is existence of the belief on looking more on the abilities by the disabled. The disagreed respondents to “CWD are able to be trained in most vocational skills” by PWND and PWD are counted for 1.1% by PWD and 5.3% by PWND, and those to statement of “CWD can learn at same rate as CWND” are 5.8% and 10.5% respectively. By these findings, it is found that respondent from PWD is less believable on the abilities of CWD in compared with those from PWND.

Other aspect of knowledge on disability is about statements of limitations by being disabled. There are three statements: (a) CWD can only participate in limited activities; (b) CWD are unable to actively move; and (c) having a disability effects to a personal intelligence. Almost answers focus agreed on statement of “CWD can only participate in limited activities”, meanwhile the disagreed responds to “CWD are unable to actively move” and “having a disability effects to a personal intelligence” are also quite high with 56.2% and 47.2% respectively.

On looking at the mean value, almost all respondents to the statements of “CWD are unable to actively moving” with 2.67 and “a disability effects to a personal intelligence” with 2.81 have their own mean value closed to the range of disagree and no ideas, meanwhile the other have close meaning to agree. Such mean statistic demonstrates the positive knowledge by respondents on areas of ability of CWD.

#### Attitudes

Evaluating the attitude in KAP module is one of the main tasks on analysing the continuity of the subject. The research defines the ways people expose their views as well as attitudes to CWD, and life conditions of CWD. The contents in this survey’s section includes: (a) Respecting: Respect should be shown to CWD, CWD should be treated like everybody else, CWD should be pity, and CWD should be ignored; (b) Having abilities: CWD needs to learn in an academic education, CWD should be given skill training, PWD work as well as PWND, CWD have more determinations than those with non-disabilities at their age, and (c) Attitudes on having supports by society: CWD should receive charity, CWD should receive charity, CWD are illness, to become a dependant is good choice for CWD, CWD should be called by their distinctive disability for the sake of remembrance, and the community have enough means to create opportunities for CWD to have social integration.

Nearly all respondents (96.2%) agree with “Respect to CWD” and also look at the positive aspects for CWD in term of abilities in studying, vocational training, social service receiving, and having a good determination. In other statements such as being dependant to other family members or society, being ignored and be called by their disability types for the sake of remembrance are disagreed mostly (Table 2).

**Table 2:** **Means’ value of statements relating to knowledge on disability**

	Statements	Means
1	Respect should be shown to CWD	4.60
2	CWD should be treated like everybody else	4.51
3	CWD do not need to learn an academic education	4.51
4	CWD should be given skill training	4.25
5	The community have enough means to create opportunities for CWD to have social integration	4.09
6	CWD should receive social services	3.88
7	CWD have more determination than those with non-disabilities at their age	3.65
8	Could PWD work as well as a person with non-disabilities	3.64
9	CWD should receive charity	3.37
10	CWD should be pitied	2.62
11	CWD are illness	2.34
12	To become a dependant is good choice for a CWD	1.99
13	CWD should be called by their distinctive disability for the sake of remembrance	1.58
14	CWD should be ignored	1.55

(ranged from 1 to 5, 1 is strongly disagreed while 5 is strongly agreed).

From this table, the statistics tell about the closet value to strongly disagree or strongly agree option for each statement. Which mean’s value closes to 5 has its meaning to be “strongly agree” and vice versa.

In recent research on disability in Vietnam (Le et al. 2008; MOLISA 2004), there is a dominant idea on calling CWD with his/her disability for the sake of remembrance, but in this survey, the respondents for such statement in option of “not agreed” are counted for 87.6%. In such responses, 100% of PWD do not agreed in comparing with 86.3% of PWND.

In analysing differences between PWD and PWND on these statements as a crosstab reference, it is found that:

- Respect to CWD is recognised with highest proportion, with 96.2%. In which, PWND who agreed is higher than that by PWD, with 96.8% and 89.5% severally. However, there is more than two time of disagreement between PWD and PWND for this statement. In other statement of “CWD should be pitted”, PWND have their own ideas on agreement higher than those of PWD, nearly 3 times (28.9% to 10.5%).
- On reviewing the statement of “CWD need to have academic education” and “CWD should be given skills’ training”, almost responds aim at education training, however more PWND agreed in the former statement (95,3% comparing 89.5%) and more PWD agreed in the latter one (89.5 in comparing with 86.3%). It is recommended that CWD have their tendencies on acquiring vocational and training skills. In other aspect, considering the determination character of CWD, which is assumed to be higher than that of CWND, among this statistics PWD have greater agreement than PWND, 78.9% in comparison with 60%.
- In recent research by UNICEF and MOLISA, one of the various recommendations for Vietnam Government is to promote and make good conditions for life of PWD due to less existed social services to them. On looking at the view of respondent on evaluating the existed community activities on assisting PWD on their social integration, disagreement of PWD to this statement is nearly two times higher than of PWND (15.8% in compared with 7.9%). In receiving the community and social services directly, PWD is aware and understanding them more comprehensively.
- There is more people focusing on CWD should receive social services than receiving charity. Meanwhile more PWD disagreed with receiving charity, there is more PWND agreed with “CWD should receiving social service”.

There are differences on responding to attitudes to disability in term of some statements, the attitudes almost aim at the positive ones and always focus on the way to have respects to PWD, to create a good conditions for PWD as well as to share the difficulties of PWD in their life.

#### Practices

This section of survey includes statements about daily activities with CWD in terms of making friend, getting along with, having permission for respondent’s kids playing, studying, making friend with, hiring CWD as worker or working with CWD. These statements are present or future probabilities. The answers for these statements are yes/no options (Table 3).

**Table 3:** **Frequency of respondents to the practice on disability (%)**

Statements	Yes (%)
Would you like to make friend with CWD/PWD?	93.3
Should CWD go to school with your child?	92.4
Could you accept a CWD to be your child’s close friend?	86.2
If you had a business, would you hire a CWD/PWD?	77.1
Have you ever given CWD money?	73.8
Would you work for a child with disabilities/people with disabilities?	60.5
Could you be a close friend to a CWD/PWD?	60.5
Would you work alongside a PWD?	43.3
If you saw a CWD on the street would you ignore him/her?	40

There are past, present and future practices toward CWD/PWD in daily life of the respondents. It seems very positive on these statements; they are expressed with high respondents. In the statement of making friend with PWD/CWD, while 100% of PWD said “yes”, just 90.3% of PWND had the same answer.

In statement of “Have you ever given CWD money?” its meaning is closed to those of statement: “CWD should receive charity”. In the latter, there is 73.8% of respondents had answered “yes”. In other statement, 40% of the respondents said yes to “If you saw a CWD on the street, would you ignore him/her”, in which PWD say “yes” higher than PWND does (52.6% in comparing with 38,9%).

Despite of the high number of respondents answering to statement of making friends with CWD/PWD, but there is lower responds relating to “be a close friend of CWD”.

There is no doubt that CWD need protection and care in daily life (UNICEF 1989), it is recommended that now is time to have more empowering and stimulating approach on taking care with CWD (MOLISA 2004). Excessive care and protection to CWD, in terms of working all for them, isolating at home which lead to social exclusion and segregation, and to economic burden as well as lost productivity (Hanoi People Committee 2011; MOLISA 2004). Like all children, CWD need to have education, assistance in order to have self-control on their life, to master their living skills that help them to take-care themselves and to have further contributions to society.

In this aspect on KAP toward disability, it is found that the practical activities, in expressing respondents’ ideas, seem very positive, but there is still a negative feedback from PWND about the daily activities of PWD. From the views on daily activities of CWD, there are still limitations on making the inclusive environments for both CWD and CWND to play together. CWD is preferred to play with CWD rather than with CWND and vice versa.

## Discussions

Vietnamese government really paid its attentions for the social situation of disability in last 20 years after the “Doi Moi” policy. The immediate applications and realisations of international approaches are so critical for dealing with problems in disability area in Vietnam. All legal documents confirm the equal rights of PWD in daily life that grounds the foundations of social awareness and social supports positively for PWD. They are ensured with their rights to equal participation in social activities, living their life with maximum independence and in social inclusion; being exempt from or paying reduced fees for social services; being provided with health care, functional rehabilitation, education, vocational training, employment, legal support, access to public buildings and transportation, information technology, and cultural, sports and tourist services as well as other services which are suitable for their type of disability and its levels.

In spite of the difference in some statements in the survey findings about the general understanding of disability, it is found that the KAP on disability is quite positive by both PWD and PWND’s views. Such social awareness plays an important key stones for making inclusive society for PWD in general and CWD in particular as well as in all social settings and in daily life. As other research findings expressed, the negative awareness is still existed in not only the research participants but also in society. This situation requires more research on promoting the social awareness on disability as well as having more practical activities and movements to change the social awareness on disability and role of PWD in society.

The definition of disability is not clear and is not mentioned directly in Vietnamese legal and policy documents including the Law on PWD (Vietnam National Assembly 2010). Almost legal documents identify the terms of PWD in general which are based on the WHO classification and its applications in Vietnamese contexts. Such approach to making the term of PWD is socially constructed and based on the social model and the medical model of disability in Vietnamese settings. The change of the name from impairment to disability in legal documents and social policies also contribute the great impacts to the welfare practice to PWD in their life. Almost law documents and social policies confirm the rights and obligations of PWD as well the responsibilities of society. These regulations are made on the directions of creating the good conditions for the individual rights and equality and responsibilities of all societal parties for making the inclusive society for all. The change of its name and contents are the significant signals to make the changes of PWD’s social status, from only support given citizens to social contributed people; from being supported to be going to support society and to be responsible for their family, their life and their society.

Approach to making up the legal and policies are based on the human rights, the Vietnamese traditional values, the international documents as well however there are lack social services to promote the policy’s efficacy in practice. The Law on PWD (Vietnam National Assembly 2010) also pointed out 10 main policy categories that imply the State’s commitments on realising the law and policies on disability in its conditions. As the understandings of disability is more preferable on the individual/medical models so there are more policies and their contents focusing on the changes for PWD rather requiring any changes of society comprehensively.

Institutionalised direction is still prominent in policies and in practice on supporting the life of PWD, especially in aspects of the health care, education and social assistance. The numbers of special institutions and special establishments for social care increased slightly in last decades which need to be reconsidered in the approach of making the inclusive society for all in policy and in practice.

From the survey findings, KAP on disability is quite positive but from previous research found the limitations of PWD in their life, and there are still limitations on social services and facilities for PWD in their daily life and in the community life which are presented in terms of education, employment, health care, transportation, cultural activities… So there is a big gap between the policy and the practice and which need the urged reform of social welfare activities for PWD.

Disability is constructed socially in Vietnamese contexts, with the traditional values of “áo lành đùm áo rách, áo rách ít đùm áo rách nhiều/People support the disabled, the disabled support the severe disabled” or “*thương ngươi như thể thương thân/support others as support ourselves*” and long-term humanity values. These values are very critical for community support and for mobilising the resources in community life. It’s also constructed based on the existed conditions of Vietnam, in approach of economic development. The welfare practice has been delivered for all people at different conditions national wide and it presents the social and human values in its activities.

Law on PWD was approved in 2010 and was effective from early of 2011. It is expected to be the significant milestone on institutionalising the State mission and strategy comprehensively on area of PWD and also on creating a favourable legal condition, equal and unrestricted conditions for the life of PWD. In addition, it is also the background for making the feasible policies and welfare practices as stated in the draft of welfare strategy to 2020: To have the welfare system for all citizens which is modern, sustainable, and suitable with the contemporary economic condition; to enlarge gradually the welfare system’s coverage and the social participation in welfare system in order to provide the entitlements of welfare polices to all vulnerable groups by the end of 2020. By the end of the strategy, all people are committed to have the minimum living standards that are suitable with the social and economic development, contributing to the mission of poverty alleviation.

## Conclusions

In order to make the feasible welfare practice for PWD in Vietnamese contexts, there are following recommendations as:

*Firstly*, it is expected to have more social activities to promote the understanding of disability that need basing on the social model and including in all social policies and practices; voice of PWD needs to be included also in the policies and strategies relating to the life of PWD.

*Secondly*, in order to implement the policies on disability in practice successfully, the way to making them must consider the voice and the requirements of PWD, and these policies should be based on inclusive approach. The inclusive approach requires the State considerations to not only PWD but also to the life of society and its condition. Further, the welfare policies are also serviced based. Welfare policies are more sustainable and benefited for PWD in the models of its potential services, in this aspect PWD need services for their further social inclusion rather than the relief social supports and assistances.

*Thirdly*, the law on PWD is the significant tool for regulating the welfare practice to PWD however in order to delivering these activities, it requires to have specific decisions and decree, circular or cross ministries and branches documents in specific areas of daily life. After more than a year of the law approve, there is not any specific document for further instruction. It’s expected to have the immediate actions and responses for the law implementations into practice. So, having the directions in policy system is very critical but to have the specific actions and services is more significant.

*Fourthly*, to promoting the health care for PWD, it is expected to develop the community based programs widely which focus on detection, intervention, and functional recovery for PWD. There is also further consideration on screening and early detecting the disability in childhood. Almost PWD live in lower living standard, so the policies on exemption and reduction of hospital fees and charges should be feasible and practical as well as providing the free health insurance cards to all PWD.

*Fifthly*, in area of education for PWD, communication should be used as a tool to enhance the social awareness of both PWD and PWND on the advantages of education, inclusive education for themselves and society. Recent recommendations from NCCD and organisations of PWD express the ideas on further developing the special education for PWD, but in inclusive approach it needs more activities, national budgets and strategy on inclusive education, so inclusive education should be the core content of education for PWD.

*Sixthly*, employment is one of significant tool for PWD realising their independence living. In order to make all favourable conditions for PWD on getting a suitable job, the vocational training model and policies are very important which need specialised basing on individuals needs and complied with the workplace demands. It is also required to have incentive policies for encouraging vocational training and on site employment for PWD.

*And finally*, other supports for PWD in terms of social supports, cultural and sport activities and accessibility in transportation and public spaces are very low quality and quantity. The State and welfare system must pay more attentions and create more policies and actions plan in these aspects in order to make the life of PWD socially included.
